# How parenthood affects the economic consequences of separation for women in same-sex and different-sex couples

**DOI:** 10.1073/pnas.2537398123

**Published:** 2026-04-22

**Authors:** Maaike van der Vleuten, Matthijs Kalmijn

**Affiliations:** ^a^Netherlands Interdisciplinary Demographic Institute-Royal Netherlands Academy of Arts and Sciences (NIDI-KNAW)/University of Groningen, The Hague 2511 CV, Netherlands; ^b^Swedish Institute for Social Research (SOFI), Stockholm University, Stockholm SE-106 91, Sweden

**Keywords:** separation, equivalized household income, same-sex couples, birth motherhood, Finland

## Abstract

Women in different-sex couples experience larger economic losses after separation than men, largely due to unequal divisions of paid and unpaid work. Women in female same-sex couples divide paid and unpaid work more equally, offering a unique opportunity to examine how partner sex, parenthood, and giving birth shape women’s economic outcomes after separation. Using Finnish population register data (2002–2020), we analyze 1,157 women in same-sex couples and 67,384 women in different-sex couples experiencing their first separation. Women in same-sex couples experience smaller reductions in equivalized household income than women in different-sex couples. Among childless women, those in same-sex couples lose substantially less income, consistent with their more equal division of labor. Among parents, women in same-sex couples also lose less than women in different-sex couples, but this difference is explained by birth motherhood. In both couple types, birth mothers lose far more income than nonbirth mothers, reflecting the concentration of caregiving and children’s residence in the birth mother’s household. Women in same-sex couples have the unique possibility to both give birth, and when they do, they experience smaller income declines after separation, indicating that more equally shared caregiving attenuates economic losses. Overall, women in same-sex couples show that more equal divisions of labor can lessen women’s losses after separation, but this advantage is overridden when caregiving and children’s residence concentrate in one household. These findings highlight the need for policies that support more equal work–care arrangements and reduce the financial costs tied to concentrated caregiving and birth motherhood.

## Significance

This study includes women in same-sex couples to advance understanding of women’s economic vulnerability after separation. Using one of the largest samples of separated women in same-sex and different-sex couples (2002–2020), we show that partners’ sex—and the associated (un)equal organization of paid and unpaid labor—shapes women’s economic losses after separation, and that these patterns shift once couples become parents. By distinguishing birth and social mothers, we reveal substantial penalties linked to birth motherhood, driven by the concentration of caregiving and children’s residence in one household. These findings extend research on postseparation inequality beyond the heterosexual context and highlight the need for policies that support more balanced work–care arrangements, particularly for birth mothers.

The economic consequences of separation are larger for women than for men in different-sex couples (DSC) (1–5). After separation, women are more likely to fall into poverty, face significant reductions in household income, and see substantial declines in their standard of living (6–8). One reason is the unequal division of paid and unpaid work established during the marriage, which reduces women’s labor market attachment and increases their financial dependence on their partner, particularly when they have children (8). By comparison, partners in female same-sex couples (SSC) are less likely to divide their paid and unpaid work (household and childcare tasks) unequally, even after having children (9–16). As such, women in SSC offer a unique opportunity to evaluate the conditions under which the economic consequences of separation are larger or smaller for women. They allow us to disentangle two processes that are typically confounded in DSC: income penalties associated with having a male partner and those associated with birth motherhood. Comparing women in SSC to women in DSC reveals the role of partner sex. Among parents, where both SSC partners are women but only one gives birth, comparisons between birth and nonbirth mothers allow us to further isolate the economic consequences of birth motherhood. Together, this approach allows us to examine how partner sex, parenthood, and birth motherhood contribute to women’s economic vulnerability after separation.

In this contribution, we examine if and to what extent women in SSC experience lower financial separation penalties than women in DSC, and how this difference is shaped by parenthood and birth motherhood. The key outcome we focus on is equivalized household income, which is a measure of individual women’s financial position that takes economies of scale and the costs of children into account (5, 17, 18). Because this measure incorporates postseparation household composition, it also captures how the allocation of children across households relates to women’s economic losses. In DSC, mothers generally experience greater separation penalties than nonmothers, in part because mothers participate less in the labor market during marriage and in part because mothers more often have children to take care of after separation than fathers (8, 19). In SSC, the role of parenthood is fundamentally different in three important ways.

First, women in SSC less often have children than women in DSC (16, 20), and this difference may explain the smaller separation penalties on average. In other words, the SSC-DSC gap in the separation penalty may be due to a compositional effect. Second, women in SSC have more equal care arrangements both before and after parenthood (12, 21) and are more likely to have joint custody (22), which may improve the financial position of both women in SSC. In DSC, mostly mothers have custody of their children, although the rise of joint custody is changing this (19, 23). Third and finally, while in DSC biological parenthood is almost always linked to the mother, birth motherhood in SSC varies within the couple type. This enables us to assess whether economic penalties after separation are associated with giving birth, rather than with being a woman partnered to a man.

There is a growing body of demographic research that focuses on documenting trends in same-sex marriage and divorce (20, 24–26), frequently finding higher dissolution rates among female SSC compared to DSC (20, 27, 28), although patterns vary by relationship duration and cohabitation status (29, 30). To our knowledge, there are, however, no studies that looked at the consequences of separation for partners in same-sex relationships, although existing research has highlighted the importance of studying these processes (31). Given the increase in the number of same-sex partnerships over the last few decades and the growing number of children being raised in same-sex households (16, 20, 32), it is time to study separation effects for nonheterosexual families. Using high-quality Finnish longitudinal register data (2002–2020), we analyze a sample of women in formalized SSC (*N* = 1,157) and DSC (*N* = 67,384) who experienced separation. Given the large scale of the data, we are able to identify sufficient numbers of women in SSC who were parents and who separated, something that is unlikely in many existing survey data. This makes it possible to provide the first evaluation of the economic consequences of separation for women and mothers in SSC.

## Finnish Context and Same-Sex Parenthood.

Finland provides an informative test case for studying the economic consequences of separation because it is characterized by high levels of gender equality, extensive welfare-state support, high rates of union dissolution, and comparatively advanced legal recognition of same-sex relationships and parenting. Comparative research shows that same-sex families are not uniformly recognized within broader definitions of “family,” and that inclusion in institutional frameworks, and thus institutional visibility, varies widely across countries (33). Against this backdrop, Finland’s comparatively early and extensive legal recognition of SSC makes its union and parenthood trajectories more consistently observable in register data. Finland is a frontrunner in the legalization of same-sex relationships, as it legalized registered partnerships in 2002 (only available for SSC, not for DSC) and marriage in 2017. Moreover, female SSC already gained access to fertility treatments in 1997, but only via private clinics not covered by the public health care system (32). Public clinics in Finland opened up to lesbian couples in 2019 (32). Second-parent adoption became possible in 2009 and joint adoption in 2017, although same-sex adoption is rare (32, 34). The relatively long period of legal recognition distinguishes Finland from many other national contexts and makes it possible to examine separation and its economic consequences among women in same-sex relationships.

One key difference between divorce and registered partnership termination is that there is a 6-mo waiting period before a divorce can be finalized, but there is no waiting period for registered partnership termination. Separation in our study is therefore defined as either the legal termination date or moving apart, whichever comes first. Separations refer to both the dissolution of registered partnerships and marriages. Note that with register data, we can only identify couples who formalized their relationship through marriage or registered partnership. In other words, our register-based definition of family follows legal recognition: It captures couples who are institutionally recorded as family units, while other women in same-sex relationships, such as cohabiting couples, may remain outside administrative records (33).

Finally, the Finnish postseparation policy context further shapes the economic consequences of union dissolution. Joint legal custody is now a common postseparation arrangement, while shared physical custody has become increasingly common relative to other countries (23, 35). At the same time, family policies largely allocate benefits to a single resident parent, while parental leave—although formally available to both parents—was structured around a primary caregiver (36–39). This combination of widespread joint custody, growing shared residence, and limited policy recognition of shared care makes Finland a distinctive setting for examining women’s economic outcomes following separation.

## Background

To examine whether women in female SSC experience lower economic penalties after separation than women in DSC, we first assess whether separation penalties differ between these groups. As outlined above, women in SSC differ from women in DSC in family formation and in how paid work, care, and child residence are organized before and after parenthood. Together, these differences suggest that women in SSC may face lower economic vulnerability following separation. Therefore, we expect:

- **H1**: Women in same-sex couples experience a smaller reduction in equivalized household income after separation than women in different-sex couples.

### The Role of Parenthood.

From research on women in DSC, we know that parenthood plays an important role in shaping the economic consequences of separation, as mothers tend to experience greater financial losses than women without children (8, 18, 40, 41). This is partly because children increase the family’s economic needs, such as housing, food, and clothing, and typically reside with their mothers after separation, raising their financial demands and lowering their equivalized household income. The rise of joint physical custody has changed this unequal postseparation burden of children but still many children primarily reside with the mother (19). In addition, children reinforce traditional divisions of paid work and care, with parents in DSC having a more gendered division of labor compared to childless women (42, 43). As a result, compared to women without children, mothers are more likely to have interrupted employment histories and reduced earnings, which increases their financial dependence on their partners during the relationship and contributes to greater income losses after separation (8, 41).

Given the prominent role of parenthood in the economic consequences of separation, differences between women in SSC and DSC may be explained by variations in the likelihood of becoming parents between the two groups. Parenthood is less common among women in SSC, often due to the relatively high financial and emotional costs associated with having children. Using Swedish register data, Kolk and Andersson (2020) found that 20% of women in same-sex marriages had children at the time of marriage, compared to 50% of women in heterosexual relationships (20). Similarly, Evertsson et al. (2025) report that approximately 70% of DSC have a second child in Denmark, Finland, Norway, and Sweden, whereas this figure is lower for SSC, ranging from 50 to 61% (16, 44). These differences in the prevalence of parenthood may contribute to variations in the economic consequences of separation between women in the two couple types. We thus expect a compositional effect:

- **H2**: Differences in equivalized household income losses after separation between women in same-sex and different-sex couples may be explained by the lower likelihood of parenthood among women in female same-sex couples compared to women in different-sex couples.

### Diverging Effects of Parenthood.

Parenthood will shape the financial consequences of separation differently for mothers in DSC and SSC. First, it is well known that parenthood increases time spent on childcare and household work, more so for mothers than for fathers in heterosexual relationships (45–47). While parenthood can also affect specialization patterns for female SSC, often with birth mothers assuming a greater share of household and childcare responsibilities, they generally maintain a more equal distribution of paid work and care after parenthood compared to parents in DSC (9–16). This more egalitarian pattern is commonly attributed to the absence of gender-differentiated roles in SSC. Because women in SSC cannot “do gender” (48) through traditionally gendered divisions of paid and unpaid work, specialization is less pronounced (12, 16, 49). Moreover, limited existing evidence indicates that SSC are more likely than DSC to adopt joint custody arrangements after separation (22), and when children are more equally divided across households, this could reduce the burden for both mothers in SSC. If this more equal division of paid work and care among female SSC forms a buffer against the economic impact of parenthood after separation, we expect the following:

- **H3**: Mothers experience a larger reduction in equivalized household income after separation than childless women.

- **H4**: Parenthood increases the reduction in equivalized household income after separation more strongly for women in different-sex couples than for women in same-sex couples.

Another factor that may influence the economic consequences of separation is birth motherhood. Because SSC include nonbirth mothers, they allow us to distinguish between birth mothers in SSC, birth mothers in DSC, and nonbirth mothers in SSC. This distinction enables us to formulate two related hypotheses.

Giving birth might lead women to be more willing to invest time in their child because of the larger physiological investment they must make during pregnancy, giving birth, and breastfeeding (50). This initial larger investment in childcare by the birth mother makes her relatively more efficient (“productive”) in taking care of the child compared to the nonbirth parent. Research from the United States shows that birth mothers in SSC spend more time on childcare, whereas nonbirth mothers spend more time in paid work (51). Similarly, in Sweden, birth mothers in SSC take nearly as much parental leave as mothers in DSC, while nonbirth mothers in SSC take significantly less, but still more than fathers (21). In Finland, birth mothers in both SSC and DSC face larger earnings parenthood penalties than their partners, indicating an unequal division of paid work and care in the years following parenthood (16). In DSC, this unequal division of labor often continues after separation, as children typically reside with their mother (19), further increasing their time spent on household and childcare tasks, limiting their ability to engage in paid work, and raising their overall household economic needs. This suggests that birth motherhood may shape women’s economic vulnerability through its role in fostering specialization during the relationship and influencing who the child lives with after separation. If this is the case, we expect birth mothers in SSC and DSC to face greater reductions in equivalized household income after separation than nonbirth mothers in SSC.

- **H5a**: Birth mothers in same-sex couples and birth mothers in different-sex couples experience a larger reduction in equivalized household income after separation than nonbirth mothers in same-sex couples.

In contrast, if parents in female SSC are more likely to divide both paid and unpaid work equally during the relationship, and to share the child’s living arrangements after separation, then time spent on caregiving, and its associated costs, are also more equally distributed. As a result, the economic burden of children could be more equally distributed between partners in SSC, and the financial consequences of separation may then be more evenly shared as well. This leads to the following additional hypothesis:

- **H5b**: Birth mothers in different-sex couples experience a larger reduction in equivalized household income after separation than birth mothers in same-sex couples.

This hypothesis is not inconsistent with Hypothesis 5a but adds nuance by suggesting a difference between birth mothers in the two union types.

## Results

We use high-quality register data including the entire population of Finland in the years 1999–2020, to identify married and registered individuals in SSC and DSC (see *Materials and Methods* section for more details). Table 1 presents descriptive statistics for women and mothers in SSC (*N* = 1,157) and DSC (*N* = 67,384) who experienced separation during this period. Women in SSC separate at an average age of 35, around 1.5 y older than women in DSC (Columns A & B). For both couple types, parents are older at separation compared to nonparents, but SSC still separate at an older age compared to women in DSC (compare Columns C & D, to G & H). Separated women in both couple types are similarly educated, but more mothers in female SSC have finished higher education (compare Columns A, B, and C to column D). Additionally, women in SSC have higher labor market earnings in the year before separation than women in DSC (Columns A & B), with an even larger gap among mothers (Columns C & D). While this is partly due to their older age at separation, it also reflects that parenthood is more selective and financially demanding for women in SSC (16). Table 1 also shows that birth mothers in SSC are younger at separation, have lower earnings, and are slightly more educated than social mothers (Columns E and F). About 16% (*n* = 63) of SSC couples had both partners give birth at least once.

**Table 1.** Descriptive statistics of separated women in same-sex and different-sex couples, by parenthood status: Means (SD in parentheses) and proportions

	Women in DSC	Women in SSC	Mothers DSC	Mothers SSC	Birth mothers in SSC	Social mothers in SSC	Childless DSC	Childless SSC
Variables	**A**	**B**	**C**	**D**	**E**	**F**	**G**	**H**
Equivalized household income^*^	27.09 (15.39)	27.92 (12.08)	25.66 (13.43)	27.06 (10.94)	26.79 (11.05)	27.51 (10.77)	30.45 (18.81)	28.36 (12.61)
Labor market earnings^*^	22.12 (19.21)	24.91 (20.44)	21.53 (19.45)	26.42 (20.35)	24.07 (20.02)	30.36 (20.38)	23.50 (18.56)	24.16 (20.45)
Own share labor market income^*^,^†^	0.39	0.50	0.36	0.50	0.45	0.59	0.45	0.51
Age at separation	33.85 (7.16)	35.41 (8.33)	34.58 (6.63)	36.59 (7.28)	36.02 (6.59)	37.55 (8.26)	32.13 (8.02)	34.80 (8.75)
Higher education (tertiary degree or higher)^*^	0.41	0.41	0.40	0.51	0.53	0.48	0.44	0.36
Registered parent with child in household 3 y before separation	0.70	0.34	1.00	1.00	1.00	–	–	–
Birth mother before separation	–	–	1.00	.63	–	–	–	–
Both mothers were birth mothers before separation	–	–	0.00	0.16	0.25	0.00		
Child < 6 y in household	0.46	0.16	0.64	0.46	0.68	0.09	0.04^‡^	0.01^‡^
Child < 18 y in household	0.61	0.20	0.85	0.58	0.86	0.10	0.05^‡^	0.01^‡^
Nr. of children in household in year of separation	1.17 (1.11)	0.30 (0.61)	1.62 (1.00)	0.81 (0.79)	1.20 (0.69)	0.15 (0.41)	0.10 (0.38)^‡^	0.05 (0.24)^‡^
Nr. of children in household year before separation^*^	1.35 (1.13)	0.51 (0.78)	1.92 (0.84)	1.43 (0.63)	1.51 (0.67)	1.30 (0.53)	0.01 (0.11)^‡^	0.04 (0.24)^‡^
Child in household in 3 y after separation	0.60	0.20	0.85	0.60	0.85	0.18	–	–
Relation duration	8.98 (5.30)	7.33 (4.78)	9.98 (5.29)	8.64 (4.58)	8.46 (4.62)	8.93 (4.51)	6.62 (4.52)	6.65 (4.74)
Repartnering after separation	0.22	0.27	0.19	0.18	0.18	0.19	0.29	0.32
Observations (individuals)	67,384	1,157	47,278	392	246^§^	146^§^	20,106	765

Abbreviations: SSC = Same-sex couples; DSC = Different-sex couples; Nr = number.

^*^Year before separation.

^†^Own share of income is 0.5 for SSC by construction, as both partners are included.

^‡^Some childless women have children in their household because they are short-term stepmothers (<1 y). Results are unchanged when recoding or excluding them.

^§^There are more birth mothers (*n* = 246) than social mothers (*n* = 146) in SSC because partners can alternate who gives birth.

**Fig. 1.**
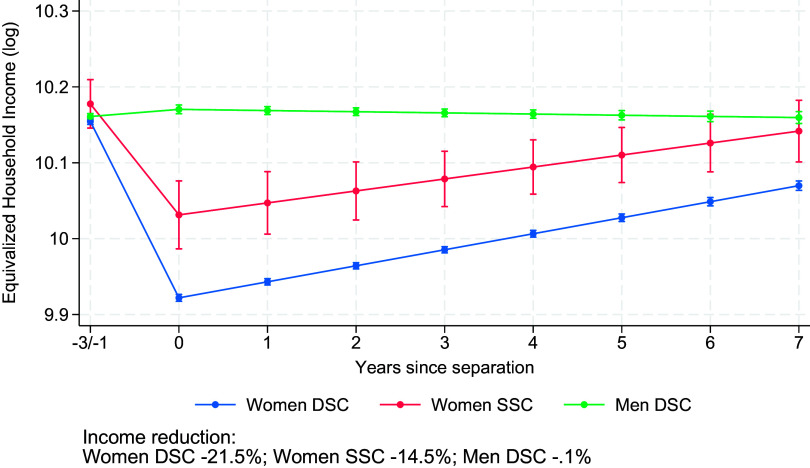
Changes in equivalized household income (log) before and after separation for women and men in female same-sex and different-sex couples. Results from random-effects models on Finnish population register data, 1999–2020 (Model 1 in *SI Appendix*, Table S1). Model-predicted values are shown for individuals aged 34 in the 2010 period and evaluated at the sample mean of education for different-sex couples. Error bars indicate 95% CI.

Fig. 1 shows the changes in the log of equivalized household income before and after separation for women in SSC and DSC (based on Model 1 in *SI Appendix*, Table S1). In line with our first hypothesis, women in DSC have a larger equivalized household income loss after separation (21.5%) than women in SSC (14.5%), with a 7-percentage-point difference. Men’s equivalized household income is not affected by separation.

**Fig. 2.**
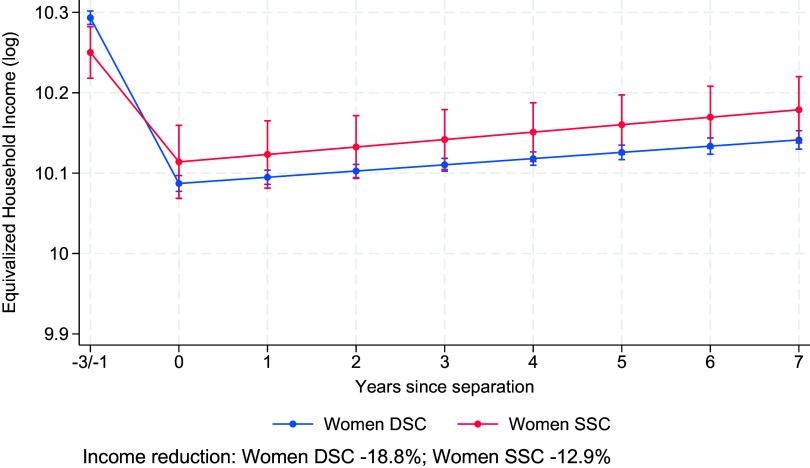
Changes in equivalized household income (log) before and after separation for women in same-sex and different-sex couples, controlled for parenthood. Results from random-effects models on Finnish population register data, 1999–2020 (Model 2 in *SI Appendix*, Table S1). Model-predicted values are shown for individuals aged 34 in the 2010 period and evaluated at the sample mean of education for different-sex couples. Error bars indicate 95% CI.

We first expected that differences in equivalized household income losses between women in SSC and DSC could be explained by the lower prevalence of parenthood among SSC (Hypothesis 2). As shown in Table 1, only 34% of separated women in SSC are parents, compared to 70% of separated women in DSC. Fig. 2 (Model 2 in *SI Appendix*, Table S1) shows that, after controlling for parenthood, the gap in equivalized household income losses decreases from 7 to 5.9% (DSC: 18.8%, SSC: 12.9%). While compositional differences in parenthood contribute to the observed gap, they do not fully account for the differences in equivalized household income losses between women in SSC and women in DSC following separation. We also checked whether *more* children contribute to larger losses, as mothers in DSC have, on average, 1.92 children in the household in the year before separation, compared to 1.43 among mothers in SSC (Table 1). We observe that the SSC–DSC gap shrinks to 3.3% (results not shown), indicating that, partly in line with our Hypothesis 2, fertility accounts for part—but not all—of the difference.

**Fig. 3.**
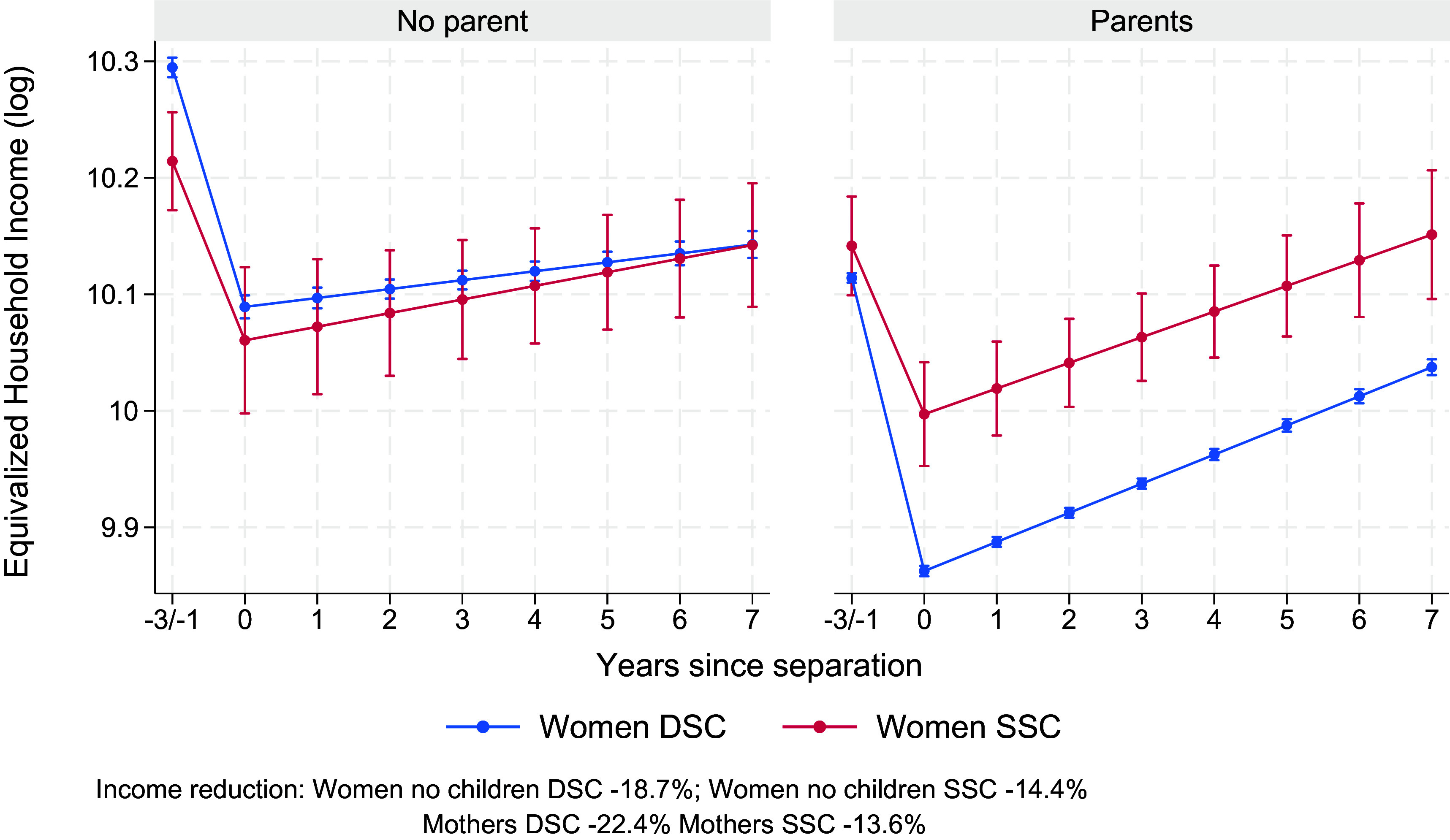
Changes in equivalized household income (log) before and after separation for women in same-sex couples and different-sex couples without children (*Left*) and with children (*Right*). Results from random-effects models on Finnish population register data, 1999–2020 (Model 3 in *SI Appendix*, Table S1). Model-predicted values are shown for individuals aged 34 in the 2010 period and evaluated at the sample mean of education for different-sex couples. Error bars indicate 95% CI.

Fig. 3 tests Hypotheses 3 and 4, namely whether parenthood exacerbates equivalized household income losses after separation (Hypothesis 3) and whether this effect is stronger for women in DSC than in SSC (Hypothesis 4; Model 3 in S1, *SI Appendix*). In line with Hypothesis 3, mothers in DSC experience larger reductions in equivalized household income (22.4%) than childless women in DSC (18.7%). In contrast, this pattern is negligible for women in SSC (childless: 14.4%; mothers: 13.6%). Thus, these findings provide clear support for Hypothesis 4: The parenthood penalty is substantially larger in DSC than in SSC. However, the expected overall parenthood penalty (Hypothesis 3) is observed only among women in DSC and not among women in SSC. This suggests that SSC’s more equal division of paid and unpaid labor and the associated “division” of children’s custody after separation may buffer parents against the larger economic consequences typically seen in DSC. A similar buffering pattern appears among childless women: Childless women in SSC experience significantly smaller losses (14.4%) than childless women in DSC (18.7%), indicating that less specialization during marriage can protect against income declines after separation.

**Fig. 4.**
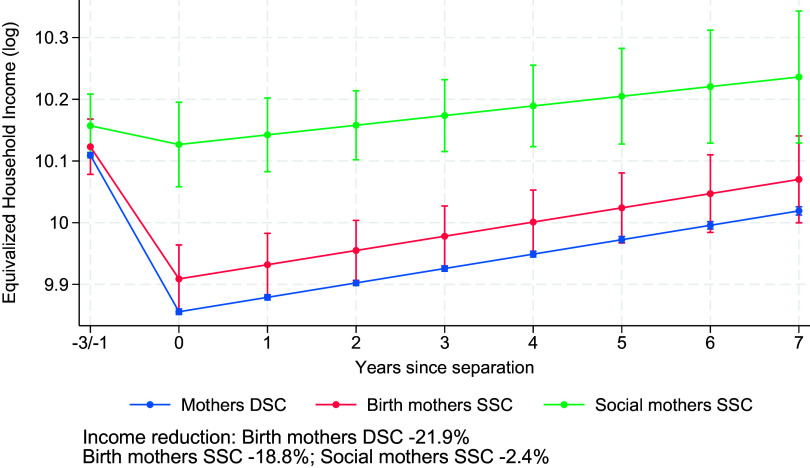
Changes in equivalized household income (log) before and after separation for birth mothers in same-sex and different-sex couples and for social mothers in same-sex couples. Results from random-effects models on Finnish population register data, 1999–2020 (Model 4 in *SI Appendix*, Table S1). Model-predicted values are shown for individuals aged 34 in the 2010 period and evaluated at the sample mean of education for different-sex couples. Error bars indicate 95% CI.

Fig. 4 shows the results assessing whether birth mothers in SSC and DSC experience greater reductions in equivalized household income than nonbirth mothers, as suggested by Hypothesis 5a, and, more specifically, whether birth mothers in DSC experience larger losses than birth mothers in SSC, as suggested by Hypothesis 5b (Model 4 in *SI Appendix*, Table S1). In line with Hypothesis 5a, Fig. 4 reveals that birth mothers in DSC and SSC lose around 21.9% and 18.8% of their equivalized household income after separation, whereas nonbirth mothers lose substantially less, around 2.4%. Although birth mothers in DSC seem to experience a slightly larger penalty compared to birth mothers in SSC, this difference was small and not statistically significant, refuting Hypothesis 5b. This pattern is due to the fact that children are registered at the birth mother’s household for both SSC and DSC. Table 1 shows that in the 3 y after separation, 85% of the birth mothers in SSC and DSC have children registered in their household, whereas this is the case for 18% of all nonbirth mothers in SSC. Looking at (nonequivalized) household income reinforces this interpretation (*SI Appendix*, Fig. B4): Household income losses are very similar for birth and nonbirth mothers (*SI Appendix*, Fig. B4), but birth mothers show much larger losses once income is equivalized (Fig. 4). This indicates that the additional penalty for birth mothers mainly reflects differences in postseparation household composition rather than larger declines in total income. Of course, the way children are divided after separation depends to a large extent on specialization during marriage, so this finding is not inconsistent with the notion of specialization penalties. Note that the strong birth motherhood effect also has compositional consequences. Because same-sex couples include more nonbirth mothers than different-sex couples, where mothers are almost exclusively birth mothers, the average separation penalty is lower for these couples than for different-sex couples.

The larger losses for birth mothers raise the question of whether losses vary by the number of children. Among birth mothers, losses are similar with one child in SSC and DSC (≈23%). For women in DSC, losses are stable across parities (22.8% with two; 20.7% with three or more), whereas for women in SSC, losses are smaller with two children (≈14.6%) (figures not shown). This pattern may reflect positive selection into higher-order births among SSC, who typically have stronger labor-market attachment and more equal earnings prior to separation, meaning that their income reduction is lower after separation.

**Fig. 5.**
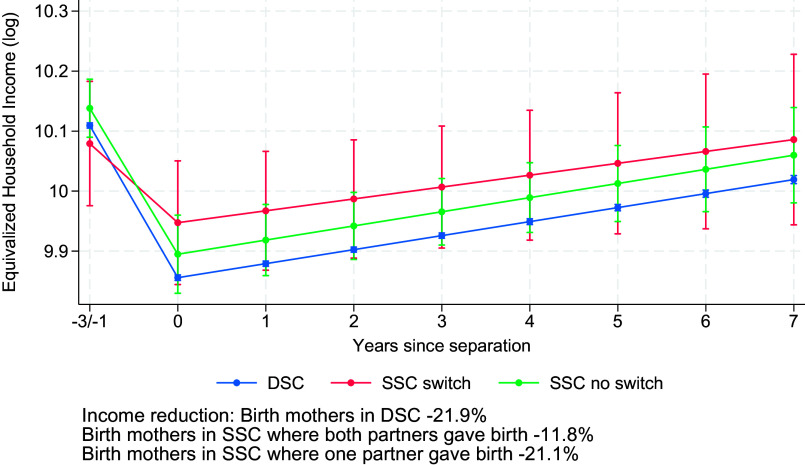
Changes in equivalized household income (log) before and after separation for mothers in different-sex couples and for mothers in same-sex couples who both gave birth and for mothers in same-sex couples where one partner gave birth. Results from random-effects models on Finnish population register data, 1999–2020 (Model 5 in *SI Appendix*, Table S1). Model-predicted values are shown for individuals aged 34 in the 2010 period and evaluated at the sample mean of education for different-sex couples. Error bars indicate 95% CI.

Last, female SSC have the unique opportunity for both partners to give birth. From qualitative studies in the Netherlands and Sweden, we know that many women plan to both carry children (52, 53), and in register data from Denmark, Sweden, Finland, and Norway, it appears that about half of the women in SSC who have a second child do take turns giving birth (16, 44). If birth motherhood is important for economic losses after separation, partners who both gave birth might divide these losses more evenly. Results of the model that examines differences between partners who both birthed a child compared to mothers who did not are displayed in Fig. 5 (Model 5 in *SI Appendix*, Table S1). The CI are large because only 16% (*n* = 63) of the parents in SSC both gave birth at least once in their lives (Table 1).

Fig. 5 shows that birth mothers in SSC who both gave birth experience an 11.8% loss in equivalized household income, compared to a 21.1% loss for birth mothers in SSC where only one partner gave birth and a 21.9% loss for mothers in DSC who carried all children. This again appears to be driven by the fact that children are often registered with the birth mother, as we see that (nonequivalized) household income losses are very similar for birth and social mothers in SSC and for birth mothers in DSC (*SI Appendix*, Fig. B5). This means that when both partners gave birth, children’s residence is more evenly split across households compared to when only one woman is the birth mother, which attenuates the income loss for each parent. While this constitutes a striking descriptive pattern, the small size of the subgroup studied here warrants caution. Power analyses indicate that at least 144 women would be required to detect statistically significant income losses at the 5% level (one-sided test). We encourage future research with larger samples to formally test these patterns.

## Discussion

By examining a group largely absent from previous research on the economic consequences of union dissolution, this paper provides novel insights into the key role of parenthood for understanding why women experience greater economic penalties after separation than men. Because the sex of the partner and birth motherhood are not contaminated in SSC, comparisons between women in SSC and DSC make it possible to distinguish income penalties associated with having a male partner from those associated with birth motherhood, mechanisms that are usually inseparable in heterosexual unions. Together, this framework reveals systematic variation in women’s economic losses after separation across family forms and clarifies the conditions under which parenthood and birth motherhood intensify or attenuate separation penalties.

We find support for our hypothesis that income reductions after separation are lower when women were in a union with another woman than when they were married to a man. We then examined the role of parenthood in accounting for these differences in three ways: compositional effects, moderation effects, and the empirical disentangling of social and biological motherhood.

First, differences in separation penalties between SSC and DSC were only partly explained by SSC’s lower parenthood prevalence, indicating that compositional differences matter but are not sufficient to account for the gap. Second, parenthood moderates this effect: Among childless couples, women in SSC experienced smaller income losses than women in DSC, consistent with prior evidence of more equal divisions of household and paid labor of SSC during marriage (12, 14). Among parents, mothers in SSC also experienced substantially smaller reductions in equivalized household income than mothers in DSC. Third, however, this difference was driven by birth motherhood. In both SSC and DSC—and thus irrespective of whether women were partnered with a man or a woman—birth mothers faced large separation penalties, whereas social mothers were much less affected by separation. These patterns indicate that separation penalties are closely tied to how children’s residence and child-related responsibilities are distributed after separation. In line with this interpretation, when concentrated caregiving was more evenly divided across households, as observed among SSC where both partners gave birth, economic penalties were smaller.

Taken together, while partners’ sex and the labor-division patterns associated with it shape women’s economic outcomes after separation, the largest penalties arise when children and caregiving responsibilities are concentrated in one household—a process shaped by pregnancy, childbirth, and institutional rules that assign children to the birth mother’s residence. This highlights that postseparation inequality is not only a consequence of gendered specialization during the union but also of how caregiving, children’s residence, and family-related resources become institutionally anchored to a single household after separation. More generally, when children are anchored to a single household after separation, that household is more likely to carry higher child-related expenses and housing demands, while likely facing tighter constraints on labor-market participation. Conceptually, this shifts the focus away from partner sex alone and toward the allocation of children and caregiving across households as a central mechanism producing women’s economic vulnerability after separation. Since specialization during marriage strongly affects how children are cared for after separation and where they reside (1, 8, 54), our evidence supports in a more general way the notion that separation penalties for women arise from an unequal division of paid and domestic labor during marriage. At the same time, our results show that even in relatively egalitarian unions, birth motherhood remains a central source of economic vulnerability.

While these findings provide important insights, several limitations should be noted. We do not observe actual custody arrangements, and parents may share care more equally than children’s registered addresses suggest. Although prior research comparing register data with survey reports suggests that the registered address generally reflects the child’s main place of residence (55), it does not fully capture shared physical custody arrangements. As a result, we may overestimate income losses for birth mothers or underestimate losses for nonresident parents. Similarly, we do not observe partners’ specialization patterns directly. Future research should explore how partners adjust their work and family roles around separation, and how these adjustments differ for birth and nonbirth mothers. Third, household composition and disposable household income are measured only at the end of the year, which limits our ability to observe month-to-month changes in household income or residence around the time of separation. This may mean that some household benefits are included in both partners’ household income, leading us to underestimate economic losses after separation. Fourth, the analysis is restricted to formalized relationships through marriage or registered partnership. As a result, nonformalized relationships are excluded. As research suggests that these relationships can differ in their separation behavior (29, 30), future research should examine separation processes in these relationships. Fifth, although the analysis covers the entire population of women in formalized same-sex unions who separated, comprising 1,157 women in SSC (including 392 parents), some subgroups remain small (e.g., SSC where both partners gave birth). Similarly, there were too few male SSC with children to be included in the analysis, which reflects the limited and often difficult pathways to parenthood available to men in SSC (56, 57). As more data become available and subgroup sizes increase, future research may be able to include these groups.

The Nordic policy context, with its extensive welfare supports and greater acceptance of LGBTQ+ families, makes Finland an informative setting for studying the economic consequences of separation. However, it may also limit the generalizability of our findings to other settings. In many countries, same-sex marriage or registered partnerships, and particularly legal pathways to same-sex parenthood, remain unavailable. As emphasized by Powell et al. (33), limited institutional recognition affects the visibility, legitimacy, and material and social support of same-sex families. This constrains the extent to which our findings can be generalized to contexts where same-sex relationships and parenthood lack legal recognition. Finland may represent a least-likely case: If substantial penalties persist even in a supportive policy environment, they may be even more pronounced elsewhere.

SSC helped reveal the conditions under which penalties are attenuated, namely, when caregiving and child-related costs are less unevenly allocated across households. Our findings therefore underscore the importance of policies that support more balanced work–care arrangements during relationships and after separation, particularly among (birth) parents. Particularly, policies that promote more equal sharing of care for children—such as nontransferable parental-leave quotas for both parents (58), or social benefits that recognize shared physical custody—may help distribute the economic costs of separation more evenly. More broadly, evidence from this unique and analytically informative group highlights how specialization and parenthood shape the gendered economic consequences of separation in the wider population.

## Materials and Methods

The Finnish register data were compiled by Statistics Finland and include information on same-sex registered partnerships from 2002–2020, same-sex marriages from 2017–2020, and different-sex marriages from 1987–2020, as well as their separation or divorce dates. We linked this information to registers on household members, sex, education, and income from various sources. Our analytical sample consists of first-time-separated women in female SSC and DSC relationships. Since same-sex legal partnerships were possible from 2002 onward, we also consider different-sex marriages that start in or after that year. Parents who jointly adopt a child are excluded. For analytical purposes, we restrict the sample to individuals who are in the data at least twice, once before separation and in the year of separation. We follow individuals from 3 y before separation to 7 y after (1999–2020). Our final analytical sample consists of 1,157 women in same-sex relationships and 67,384 women in different-sex relationships.

### Variables.

*Equivalized household income* is created by first taking the sum of each household member’s disposable annual household income (inflation-adjusted using the consumer price index, reference year is 2014), based on registered household membership. Disposable money income consists of gross income (sum of earned income, entrepreneurial income, property income, and current transfers received) after taxes and other levies. Negative values in the underlying income components are bottom-coded at zero prior to constructing household income. We adjust this measure for household size, and more particularly, the presence of the partner and the number of children in postseparation households, using the OECD modified equivalence scale. This gives the first adult in the household a weight of 1; subsequent adults are given a weight of 0.5, and each child below 14 y of age is given a weight of 0.3. Note that this measure is based on children’s residence rather than custody arrangements. The equivalence scale thus enables comparisons between households of different sizes and compositions. We take the natural logarithm of equivalized household income to reduce skewness in the distribution, and add one euro prior to logging to retain individuals with zero disposable household income.

*Couple type* refers to being in a same-sex or different-sex relationship, as identified by the registered legal sex of the partners.

*Separation event* is a dummy variable that takes a value of 0 for the years before the separation and 1 from the year of separation onward.

*Recovery time* reflects the postseparation recovery period. It takes the value of 0 for the years before and the year of separation and then follows a linear progression in subsequent years.

*Parent* refers to an individual officially registered as a child’s parent and has lived with that child for at least 1 y during the 3 y preceding the separation.

*Birth parent* is a dummy that indicates whether mothers ever gave birth to a child before the year of separation (1) or not (0). These mothers are also legally registered parents and have lived with the child in at least one of the three years preceding separation.

*Age* is measured in years, and *calendar time* is a categorical variable that represents calendar years in the following intervals: 1999, 2000/2004, 2005/2009, 2010/2014, and 2015/2020.

The highest education level is a categorical indicator coded as *higher education*, including bachelor’s or equivalent level, master’s or equivalent level, doctoral or equivalent level, and *low to medium level* education. Missing data were coded as having lower education for individuals born in Finland because Statistics Finland does not register primary education. However, it is reasonable to assume that people born in Finland have at least primary education (9.9%). The remaining missing cases (2.8%) were also coded as lower education to include them in our analyses. Excluding either of these groups of missing cases did not affect our conclusions.

We do not control for earnings because our outcome already incorporates women’s earnings, and conditioning on a component of the outcome would bias estimates. However, controlling for the level of earnings for partners in SSC and DSC in the 2 y before separation showed highly similar results and did not affect our conclusions.

### Empirical Strategy.

The time window we focus on is from 3 y before separation to 7 y after separation. The general design we use is based on the so-called step-impact function (59), where the income in each year since separation is compared to the income in the years before separation. The main advantage of this approach is that separate parameters can be obtained for the initial decline after separation and the subsequent recovery after the initial loss (18).

We applied random-effects models to the yearly register data, accounting for observations nested within individuals. Since everyone went through a separation at some point during the observation period, separation is not associated with any unmeasured, stable characteristics of individuals over time (60). Consequently, the fixed-effects estimator’s ability to reduce this bias is not applicable. Moreover, random-effects models are appropriate in our case because we are interested in explaining variance *between* women in SSC and DSC, variation that fixed-effects models eliminate by construction.^*^

Five main models were estimated. Model 1 interacts couple type with the separation event variable to estimate the initial income drop for women in SSC and DSC (H1), and further interacts couple type with the recovery time to fully capture potential differences in recovery trajectories between the two groups. To examine whether differences in income losses between women in SSC and DSC can be attributed to parenthood (H2), Model 2 adds interactions between parenthood and separation event as well as between parenthood and recovery time. Model 3 estimates whether the economic consequences of separation differ jointly by parenthood status and couple type. To do so, it includes three-way interactions between the separation event, couple type, and parenthood, as well as between recovery time, couple type, and parenthood. This specification allows us to test whether the economic consequences of separation are larger for mothers than childless women (H3), and whether this penalty differs between SSC and DSC (H4). Model 4 interacts birth motherhood with the separation event and recovery time to evaluate how income losses vary among birth mothers in SSC, birth mothers in DSC, and non–birth mothers in SSC (H5a/H5b). Model 5 interacts the separation event and recovery time indicator with a variable distinguishing birth mothers in DSC (0), birth mothers in SSC where both partners gave birth (1), and birth mothers in SSC where only one partner gave birth (2). All models control for age, age squared, and calendar time as main effects but not in interaction with the separation variables. Because education may affect the size and pace of income changes after separation, both the main effect of education and its interaction are included. Age and calendar time are included only as basic controls. Because women in SSC are clustered within couples, SE are clustered at the couple level.

## Supplementary Material

Appendix 01 (PDF)

## Data Availability

The data used in this study are derived from Finnish population register data and cannot be shared publicly. Researchers can apply for access to these data through Statistics Finland. Information on the application process, access conditions, and contact details is available at Statistics Finland’s research services website (https://stat.fi/en/services/services-for-researchers) (61). This study was conducted under a data permit granted by Statistics Finland (permit number TK/1494/07.03.00/2023), in accordance with national data protection regulations. Based on the review procedures of Statistics Finland, separate institutional ethics committee approval was not required. Informed consent was not required because the study uses deidentified administrative register data covering the population, for which obtaining individual consent is not feasible, and which are made available for research under national statistical legislation and an approved data permit. Code to reconstruct the analyses is available at 10.17605/OSF.IO/R6SJ3 (62).

## Footnotes

^*^As a robustness check, we did estimated individual fixed-effects models, including a comparison group that remains married. Fixed effects remove all time-invariant differences between individuals, and the specification (with age and period controls) separates changes associated with separation from general life-cycle and period trends. However, because fixed effects differ away from the between-person SSC–DSC contrast we seek to explain, Model 2 (Fig. 2) is not estimated with fixed effects. The fixed-effects results are therefore shown in the *SI Appendix*, Figs. A1 and A3–A5, and the patterns, and therefore conclusions, are very similar.
