# Supplementary material for: How parenthood affects the economic consequences of separation for women in same-sex and different-sex couples
Source: Proc Natl Acad Sci U S A. 2026 Apr 22;123(17):e2537398123. doi: 10.1073/pnas.2537398123 (PMC13123900; doi:10.1073/pnas.2537398123)
Supplement: Supplementary file 1 — Appendix 01 (PDF) [file pnas.2537398123.sapp.pdf]

SI Appendix.

Table S1. Random-effects models estimating changes in equivalized household income around separation for women and mothers in same-sex couples (SSC) and different-sex couples (DSC) (Models 1–5).

	Model 1	Model 2	Model 3	Model 4	Model 5
Separation event (before separation, ref.)	-	-	-	-	-
Separation event	-0.242***	-0.208***	-0.207***	-0.248***	-0.248***
	(0.00250)	(0.00518)	(0.00522)	(0.00264)	(0.00264)
Women in DSC (ref.)	-	-	-	-	-
Women in SSC	0.0231	-0.0431**	-0.0805***		
	(0.0164)	(0.0164)	(0.0218)		
Men in DSC	0.00642***				
	(0.00135)				
Separation event × women in DSC (ref.)	-	-	-	-	-
Separation event × women in SSC	0.0863***	0.0700***	0.0518*		
	(0.0153)	(0.0154)	(0.0219)		
Separation event × men in DSC	0.242***				
	(0.00328)				
Childless women (ref.)		-			
Parent		-0.179***	-0.181***		
		(0.00408)	(0.00412)		
Parent × separation event		-0.0451***	-0.0461***		
		(0.00497)	(0.00504)		
Women in SSC × parent			0.108***		
			(0.0303)		
Separation event × women in SSC × parent			0.0554*		
			(0.0273)		
Mothers in DSC (ref.)				-	-
Birth mothers in SSC				0.0137	
				(0.0229)	
Social mothers in SSC				0.0478	
				(0.0261)	
Separation event × birth mothers in SSC				0.0395	
				(0.0231)	
Separation event × social mothers in SSC				0.223***	
				(0.0257)	
Both partners gave birth in SSC					-0.0302
					(0.0529)
One mother gave birth in SSC					0.0288
					(0.0247)
Separation event × both partners gave birth in SSC					0.122***
					(0.0294)
Separation event × one mother gave birth in SSC					0.0103
					(0.0289)
Recovery time (pre-separation and year of separation)	-	-	-	-	-
Recovery time (years after separation)	0.0216***	0.00782***	0.00771***	0.0231***	0.0231***
	(0.000609)	(0.00117)	(0.00117)	(0.000642)	(0.000642)
Women in SSC × recovery time	-0.00534	0.00152	0.00404		
	(0.00340)	(0.00342)	(0.00443)		
Men in DSC × recovery time	-0.0227***				
	(0.000755)				
Parent × recovery time		0.0172***	0.0174***		
		(0.00108)	(0.00110)		
Women in SSC × parent × recovery time			-0.00702		
			(0.00655)		
Birth mothers in SSC × recovery time				-0.000308	
				(0.00562)	

Social mothers in SSC × recovery time				-0.00771	
				(0.00954)	
Both partners gave birth in SSC × recovery time					-0.00356
					(0.00976)
One mother gave birth in SSC × recovery time					0.000223
					(0.00667)
**Controls**					
1999	-0.229**	-0.225**	-0.225**	-0.169	-0.168
	(0.0715)	(0.0804)	(0.0804)	(0.119)	(0.119)
2000-2004	-0.0953***	-0.115***	-0.115***	-0.125***	-0.125***
	(0.00457)	(0.00544)	(0.00544)	(0.00606)	(0.00606)
2005-2009	-0.0218***	-0.0398***	-0.0398***	-0.0526***	-0.0525***
	(0.00229)	(0.00267)	(0.00267)	(0.00280)	(0.00280)
2010-2014 (ref.)	-	-	-	-	-
2015-2020	0.0180***	0.0132***	0.0132***	0.0177***	0.0178***
	(0.00162)	(0.00187)	(0.00187)	(0.00180)	(0.00181)
Age	0.0639***	0.0698***	0.0698***	0.0605***	0.0605***
	(0.00115)	(0.00138)	(0.00138)	(0.00154)	(0.00155)
	-	-	-	-	-
Age × age	0.000752***	0.000819***	0.000819***	0.000678***	0.000679***
	(0.000015)	(0.000019)	(0.000019)	(0.000021)	(0.000021)
Low to medium education (ref.)	-	-	-	-	-
Higher education (Tertiary or more)	0.239***	0.232***	0.231***	0.213***	0.213***
	(0.00282)	(0.00341)	(0.00341)	(0.00369)	(0.00369)
Separation event × higher education (Tertiary or more)	0.0236***	0.00475	0.00459	-0.0142***	-0.0145***
	(0.00317)	(0.00390)	(0.00390)	(0.00399)	(0.00400)
Recovery time × higher education (Tertiary or more)	-0.00123	-0.000178	-0.000152	0.000580	0.000588
	(0.000771)	(0.000905)	(0.000905)	(0.000929)	(0.000930)
Constant	8.751***	8.773***	8.773***	8.750***	8.749***
	(0.0207)	(0.0239)	(0.0239)	(0.0270)	(0.0271)
N (person-years)	1079603	553318	553318	378412	377356
N individuals	134,235	68,541	68,541	47,670	47,524

Notes: Coefficients are from random-effects regression models estimated on longitudinal Finnish population register data. The unit of observation is the person-year. SSC denotes same-sex couples and DSC denotes different-sex couples. Standard errors are reported in parentheses. * $p < 0.10$, ** $p < 0.05$, *** $p < 0.01$.

Model 1 interacts couple type with separation event and recovery time to estimate differences between SSC and DSC in the initial income drop and subsequent recovery.

Model 2 adds interactions between parenthood and separation event and recovery time to assess whether parenthood accounts for differences between SSC and DSC.

Model 3 includes three-way interactions between couple type, parenthood, and separation event, as well as between couple type, parenthood, and recovery time, to examine whether the effect of parenthood on income losses differs between SSC and DSC.

Model 4 interacts birth motherhood with separation event and recovery time to compare birth mothers in SSC, birth mothers in DSC, and non–birth mothers in SSC.

Model 5 interacts separation event and recovery time with a variable distinguishing birth mothers in DSC, birth mothers in SSC where both partners gave birth at least once, and birth mothers in SSC where only one partner gave birth.

All models control for age, age squared, calendar period, education (the latter as interactions between education and the separation event and recovery time).

A. Fixed effects models including the married population.

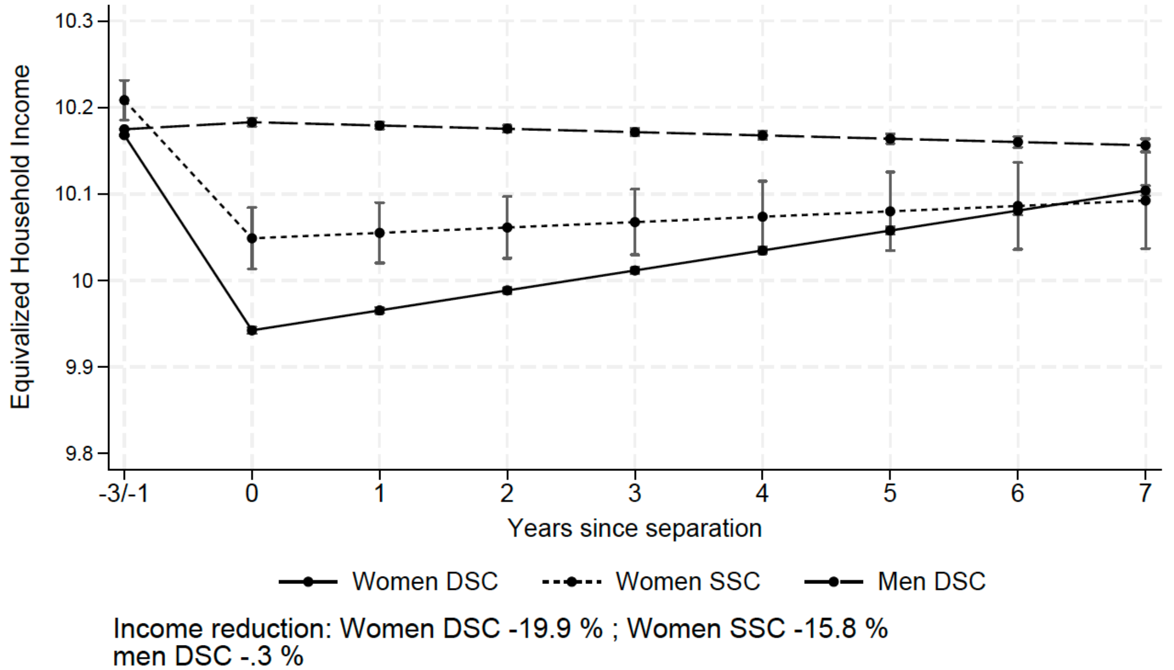

Fig. A1. Changes in equivalized household income (log) before and after separation for women and men in female same-sex and different-sex couples. Results from fixed-effects models on Finnish population register data, 1999–2020. Model-predicted values are shown for individuals aged 34 in the 2010 period and evaluated at the sample mean of education for different-sex couples. Error bars indicate 95% confidence intervals.

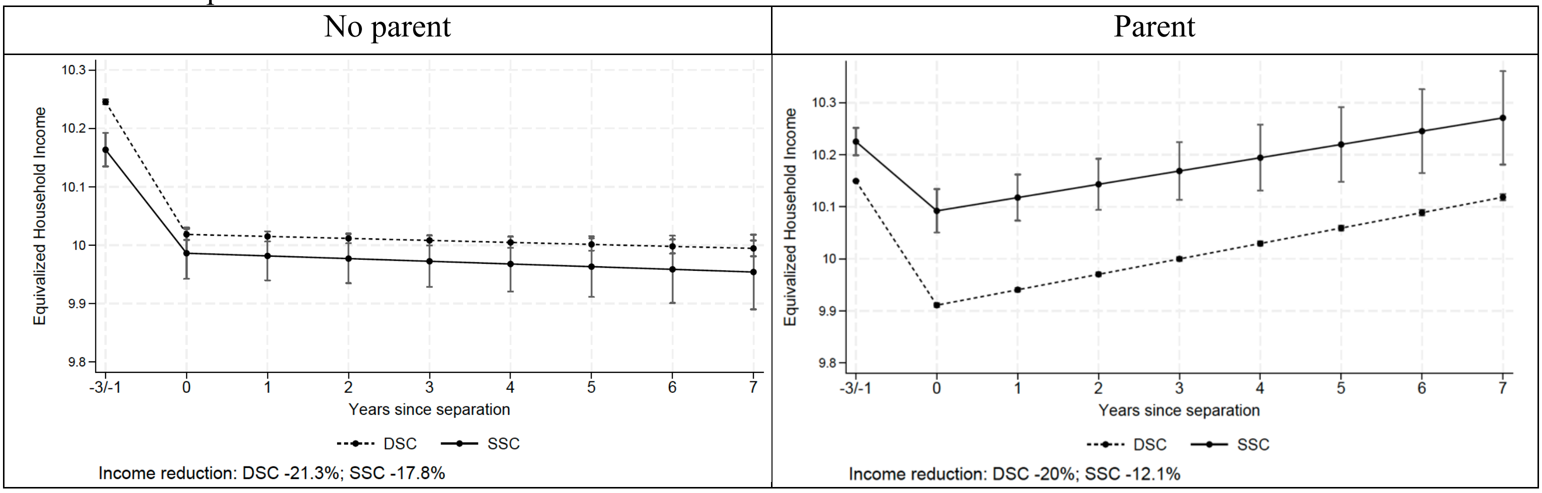

Fig. A3. Changes in equivalized household income (log) before and after separation for women in same-sex couples and different-sex couples without children (left panel) and with children (right panel). Results from fixed-effects models on Finnish population register data, 1999–2020. Model-predicted values are shown for individuals aged 34 in the 2010 period and evaluated at the sample mean of education for different-sex couples. Error bars indicate 95% confidence intervals.

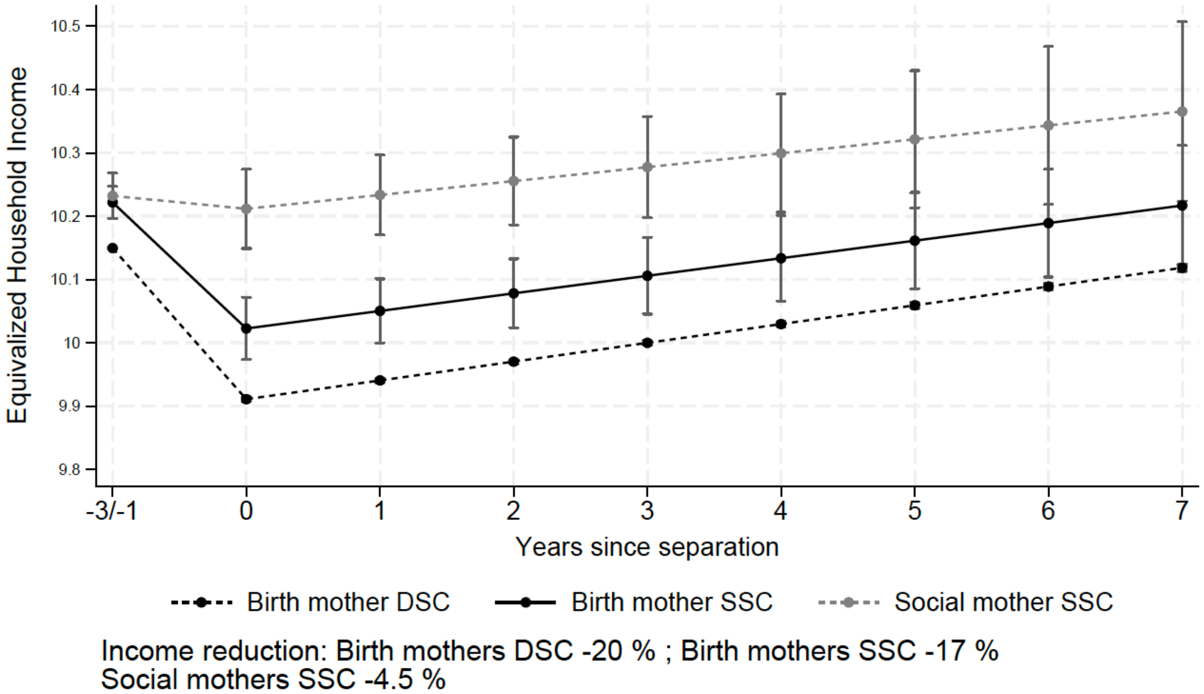

Fig. A4. Changes in equivalized household income (log) before and after separation for birth mothers in same-sex and different-sex couples, and for social mothers in same-sex couples. Results from fixed-effects models on Finnish population register data, 1999–2020. Model-predicted values are shown for individuals aged 34 in the 2010 period and evaluated at the sample mean of education for different-sex couples. Error bars indicate 95% confidence intervals.

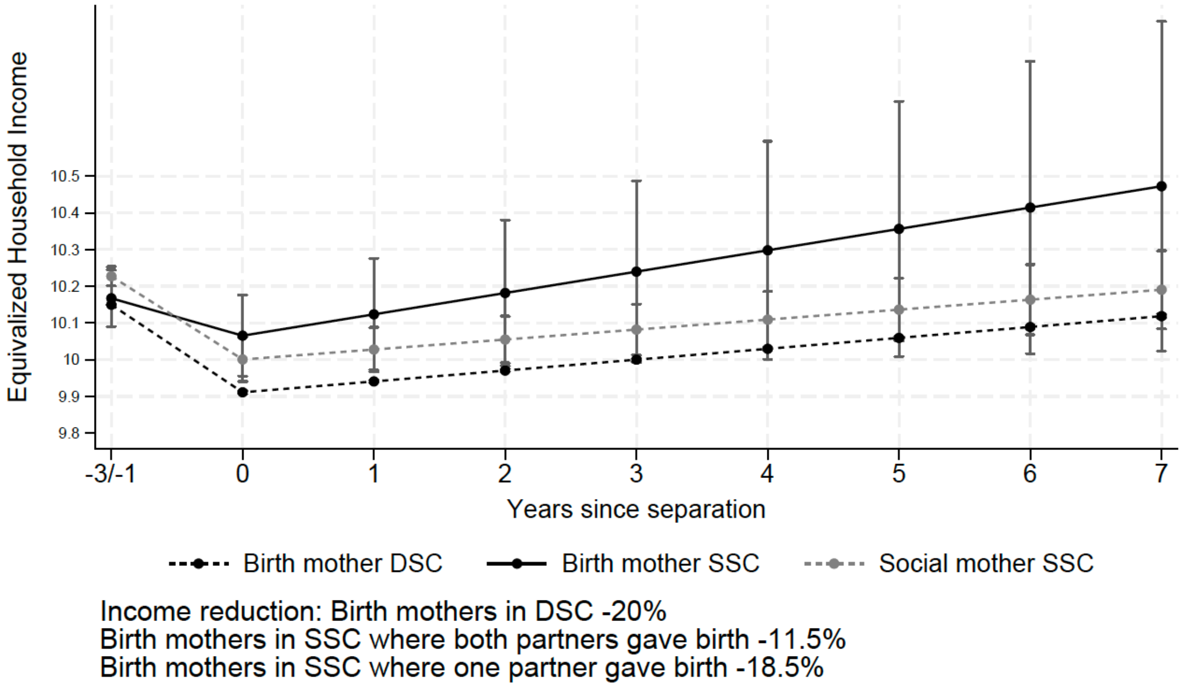

Fig. A5. Changes in equivalized household income (log) before and after separation for mothers in different-sex couples and for mothers in same-sex couples who both gave birth and for mothers in same-sex couples where one partner gave birth. Results from fixed-effects models on Finnish population register data, 1999–2020. Model-predicted values are shown for individuals aged 34 in the 2010 period and evaluated at the sample mean of education for different-sex couples. Error bars indicate 95% confidence intervals.

B. Household income

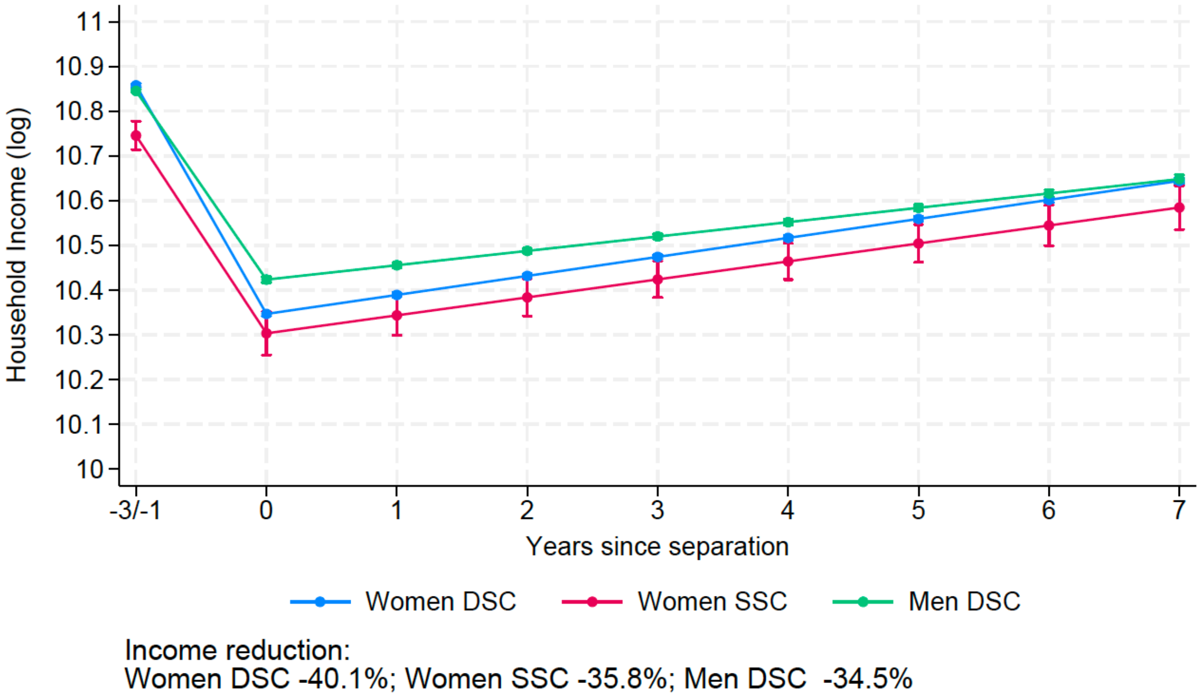

Fig. B1. Changes in household income (log) before and after separation for women and men in female same-sex and different-sex couples. Results from random-effects models on Finnish population register data, 1999–2020. Model-predicted values are shown for individuals aged 34 in the 2010 period and evaluated at the sample mean of education for different-sex couples. Error bars indicate 95% confidence intervals.

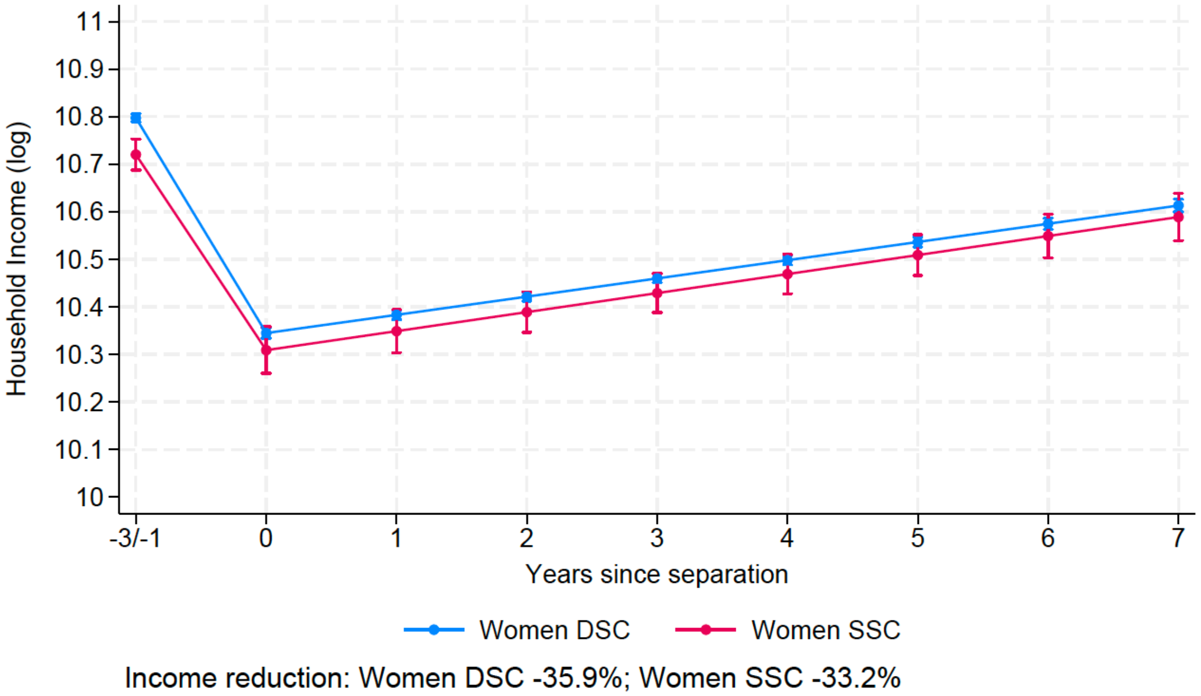

Fig. B2. Changes in household income (log) before and after separation for women in same-sex and different-sex couples, controlled for parenthood. Results from random-effects models on Finnish population register data, 1999–2020. Model-predicted values are shown for individuals aged 34 in the 2010 period and evaluated at the sample mean of education for different-sex couples. Error bars indicate 95% confidence intervals.

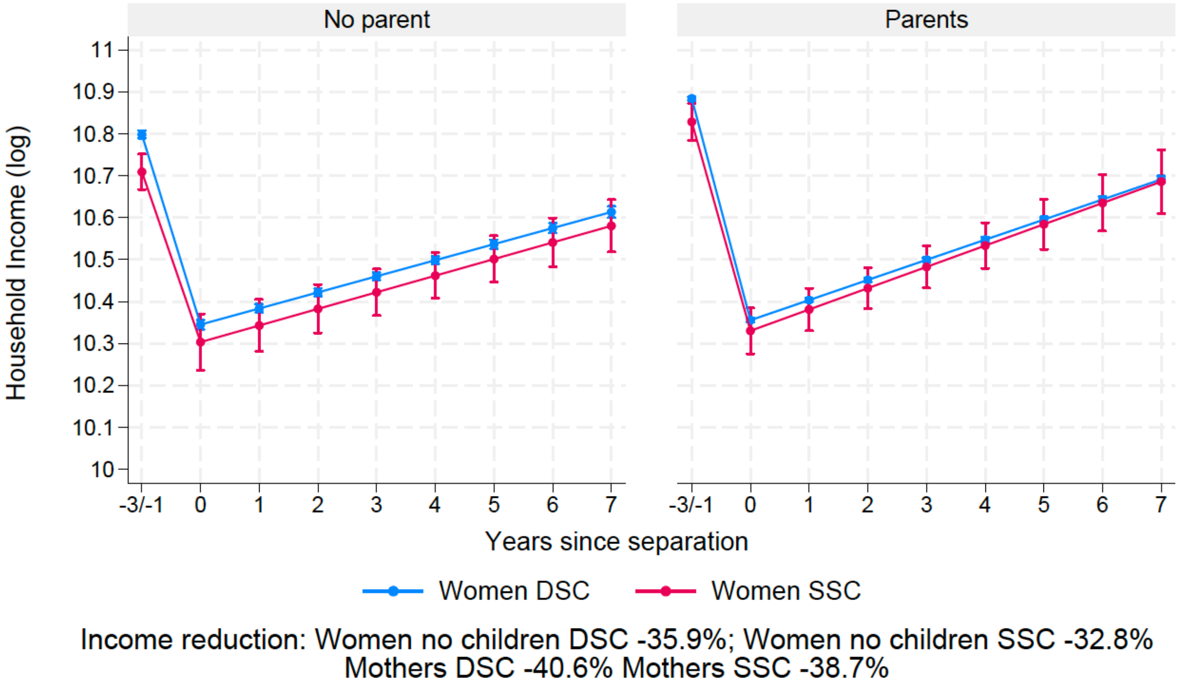

Fig. B3. Changes in household income (log) before and after separation for women in same-sex couples and different-sex couples without children (left panel) and with children (right panel). Results from random-effects models on Finnish population register data, 1999–2020. Model-predicted values are shown for individuals aged 34 in the 2010 period and evaluated at the sample mean of education for different-sex couples. Error bars indicate 95% confidence intervals.

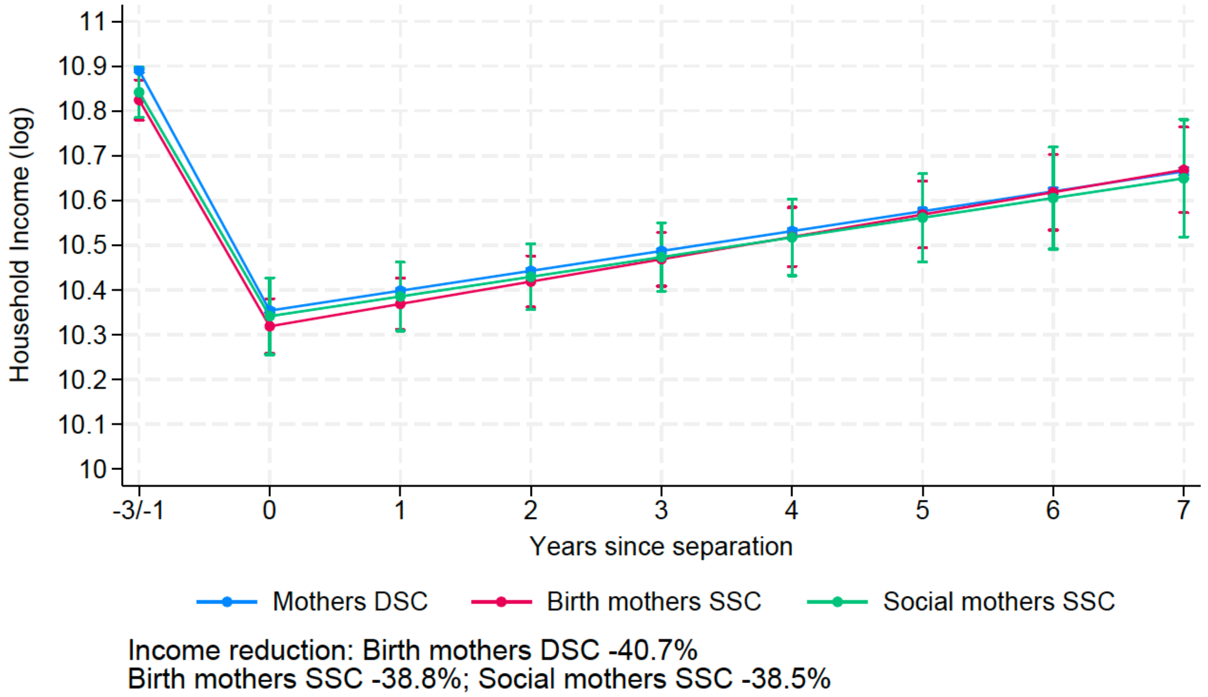

Fig. B4. Changes in household income (log) before and after separation for birth mothers in same-sex and different-sex couples, and for social mothers in same-sex couples. Results from random-effects models on Finnish population register data, 1999–2020. Model-predicted values are shown for individuals aged 34 in the 2010 period and evaluated at the sample mean of education for different-sex couples. Error bars indicate 95% confidence intervals.

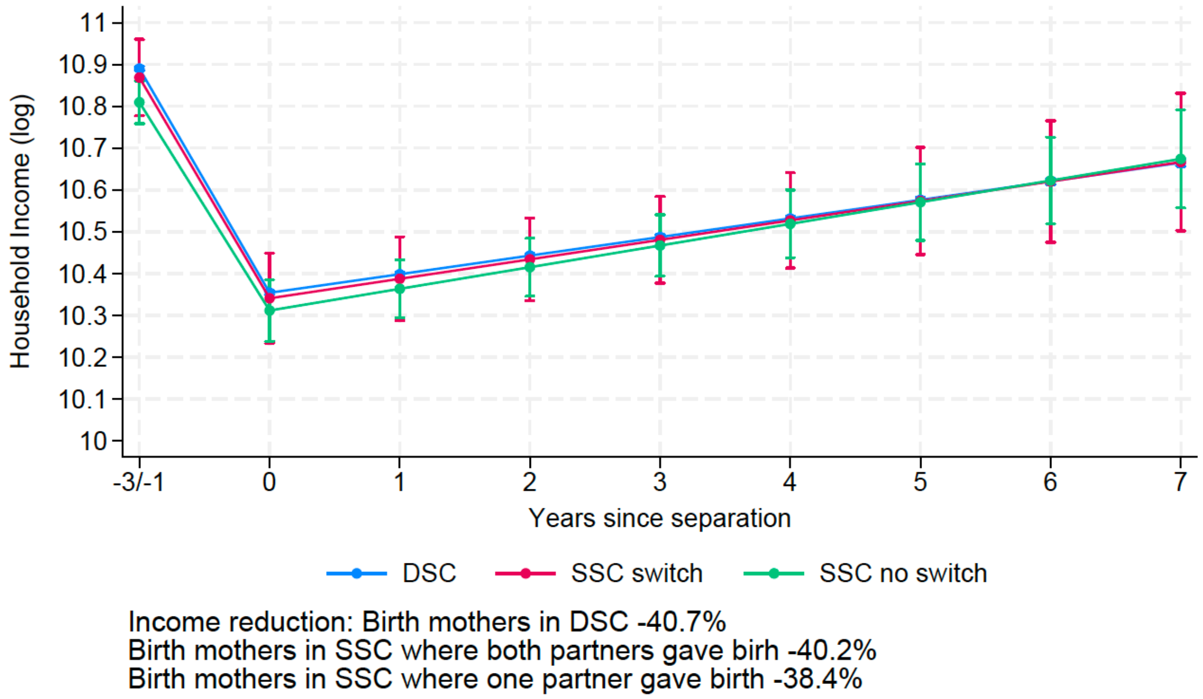

Fig. B5. Changes in household income (log) before and after separation for mothers in different-sex couples and for mothers in same-sex couples who both gave birth and for mothers in same-sex couples where one partner gave birth. Results from random-effects models on Finnish population register data, 1999–2020. Model-predicted values are shown for individuals aged 34 in the 2010 period and evaluated at the sample mean of education for different-sex couples. Error bars indicate 95% confidence intervals.
